# Challenges facing modern automated laboratories' robotic applications

**DOI:** 10.1155/S1463924691000056

**Published:** 1991

**Authors:** Elsayed A. Elnenaey, Josip Pluscec, Vincent Fernandez

**Affiliations:** Bristol-Myers Squibb Technical Operations North America Quality Control/Quality Assurance PO Box 191 New Brunswick New Jersey 08903 USA


Journal of Automatic Chemistry, Vol. 13, No. 1 (January–February 1991), pp. 17–21

### Challenges facing modern automated laboratories' robotic applications*

**Elsayed A. Elnenaey, Josip Pluscec and Vincent Fernandez**

*Bristol-Myers Squibb Technical Operations, North America Quality Control/Quality Assurance, PO Box 191, New Brunswick, New Jersey 08903, USA*

### Introduction

The pharmaceutical industry has a growing need for efficient analytical laboratories that can keep up with the increasing workload generated by other areas within the industry. The increased sample throughput and quick turnover of analytical data are key factors for speedy new product approval by regulatory agencies. To keep up with the continuous increase in workload generated for these laboratories, full automation is determined to be an unavoidable task. This applies to instrumentation, computers and data management, as well as to sample preparation. Efficiency in chemical laboratories is greatly improved by applying automated sample preparation technology. Automated sample preparation via robotics also improves safety and minimizes exposure of personnel to hazardous materials, which helps to achieve Good Laboratory Practice (GLP).

The process of acquiring a laboratory robot may differ from one organization to another. The first-time acquisition of a laboratory robot is the most critical, where the outcome of the event will greatly affect future decisions. Based on experience within New Brunswick facility, the different stages of the decision-making process and the start-up procedure of a laboratory robotic system are discussed. Typical examples of various applications within the pharmaceutical industry are presented.

### The process of acquiring robotic system

The following is a list of the important issues to be addressed during this process:

(1) Initial study.

(2) Justification.

(3) Selecting and designing the robotic system.

(4) Implementation.

(5) Validation.

### *Initial study*

After nearly 8 years since the introduction of robotic technology, some laboratory personnel and managers still have doubts about the reliability of laboratory robots, while many others have become more comfortable with the technology and the degree of sophistication it achieved. Laboratory robots have provided the means to automate a variety of applications, especially within the pharmaceutical industry. Robots, like many other sophisticated instruments, are not 100% failure immune. However, it is important to understand the causes in order to minimize and prevent failures. Laboratory robots provide, through successful applications, reliable, accurate and cost-effective alternatives to manual operations. In a typical analytical laboratory, there are conventional high-volume products and/or tests. These are the ones that should be targeted first for automation. Estimated cost savings may be calculated based on manual preparation time, and the maximum number of samples per analyst/shift versus robotic time and maximum number per 24 h period. An additional feature would be a multi-application robot that can handle more than one test. If there are not enough samples of one application to keep the robot occupied 7 days a week, the alternative will be to run accumulated samples for application 1, then application 2 to follow and so on. The length of the cycle will depend on the number of samples for the particular application. Other factors that may be considered during the initial study are: (i) the complexity of the test, (ii) common steps that might be shared with other applications, and (iii) environmental impact. The conclusion of this stage should present a comparison of current status, e.g. the total test hours, maximum number of samples tested per shift, the overall retest rate, the environmental aspects and safety of personnel, versus the proposed robotic operation.

### *Justification*

The project leader should be dedicated, committed and should understand the concept of laboratory automation in order to communicate, troubleshoot and answer all questions about the proposed applications.

The following are some guidelines to be considered throughout the process of project justification.

(1) The importance of the application to the short- and long-term planning of the organization.

(2) The improved quality of testing.

(3) Increased productivity and efficiency.

(4) Cost effectiveness; most applications will offer a pay back period of about 3–4 years.

(5) The possibility of using the proposed robotic system to perform more than one test.

* Abstract published in *Journal of Automatic Chemistry*, Vol. 12, No. 6.

(6) General cleanliness and better implementation of GLPs.

(7) Safety of personnel from exposure to hazardous chemicals and procedures.

(8) Are there other robots owned by the organization? Who is the manufacturer and how successful they are in their applications? This may create an opportunity for exchange of valuable experience within the organization.

*The process of selecting and designing the system*

The system design and layout is a very critical step. This includes thorough knowledge of the available modules that duplicate the manual procedure. A perfect match is often not possible, so certain alternatives should be explored, e.g. redeveloping the analytical method, approaching the vendor for possible manufacturing of special hardware components. The following example represents a typical study conducted for the recent acquisition of new robot by chemical control department at Bristol-Myers Squib, New Brunswick.

(1) A review of the manual analytical method will serve as starting point for generating a list of robotic modules to automate the method.

(2) A sketch of the bench-top layout plan will be used to simulate the different steps of the application.

(3) Occasionally, a problem may arise due to the fact that a custom-designed module is not yet available on the market. These cases should be isolated and addressed separately with the vendor for the possibility of special manufacturing and/or modification of the method.

(4) Necessary information should be available about the compatibility of existing equipment that might be used to complement the robot.

(5) Initiation of technical discussions with vendors. The outcome of such communication should reflect the best available hardware/software combination to handle the subject application. A bench-top layout should be finalized at the conclusion of this stage. Also, a reliable estimate for the overall cost of the robotic system and the necessary accessories should be determined at this time. It is important to understand that the total project budget would be more than just the robot cost. Examples of the extra cost related to the project: site preparation, e.g. clean power line, sufficient outlets, adequate utilities, waste disposal, inert gas supplies, removing or adding laboratory benches or tables, adequate ventilation for quick removal of solvent vapors, etc. The cost of programming the specific application is always an option on top of the purchase price, and the decision on that should be made on a case-by-case basis depending on available resources and funds.

This process of selecting a robot is quite lengthy especially for new and/or very specialized applications. Extensive communications with vendors proved to be very valuable during this period.

*Implementation stage*

This process starts after the purchase order has been sent out. Co-ordination between delivery of the robotic hardware, developing application software, personnel training, delivery of other instruments (e.g. HPLC, GC, dissolution system) should be expedited and monitored very closely. Appropriate timing in receiving all parts and accessories are crucial for a successful start. If the robot is designed to perform simple routines, such as Karl Fischer and weight variation, the start-up time and validation process usually require less time than other complex applications. As a guideline, for multi-application robotic systems, it is recommended to start with the less complex application, to complete it and start accumulating actual savings early in the process. This also has some advantage in boosting personnel morale and management confidence.

*Validation*

The validation process should start right after complete installation. Dry runs and real preliminary trials are highly recommended. A validation protocol should be approved by Quality Assurance or the regulatory authority within the organization.

Based on the approved guidelines in the validation protocol, the process should begin by challenging both hardware and software robotic commands. Examples may include alphanumeric inputs, proper sample and standard sequence, balance accuracy, Master Lab Station accuracy, solvent and column switching valve selections, interface to laboratory information system and data management, waterbath temperature, and proper controlling of other equipments and peripherals. The second stage of the initial validation may involve direct comparison of manual versus robotic data. When the final validation report is approved, the robotic system can be used for routine testing. The validation should not stop at the initial installation stage; all the critical parameters of the application (e.g. solvent delivery, balance check, vortexing time, ultrasonic bath efficiency, etc.) should be checked periodically according to a well-established schedule. It is probably a good idea to have a special software program to perform critical parameters on predetermined intervals where the mechanical moves and other parameters can be checked by an analyst. Any slight deviation of the preset parameters should be corrected and revalidated immediately. A preventative maintenance program is highly recommended.

### Typical examples of robotic applications in pharmaceutical laboratories

The following examples represent various applications within the Bristol-Myers Squibb (BMS), New Brunswick location.

The Zymate II system in figure 1 is used by Quality Control for tablet content uniformity testing. The system can test five lots of four different cardiovascular products (10 tablets each) in one single run. It is equipped with an HPLC column-switching valve and a mobile phase-

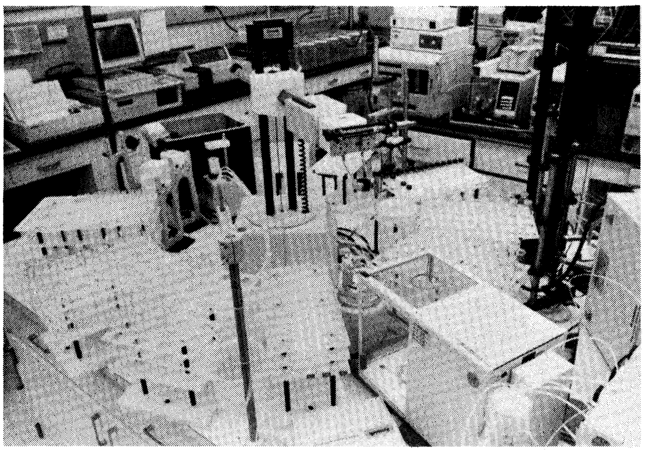

*Figure 1. A Zymate II robotic system for tablet content uniformity testing by HPLC.*

switching valve for automatic selection of the appropriate condition for each chromatographic run of each product. An additional second dilution step for higher potency is automatically selected by the program when the appropriate product code is inputted. Same-day manual testing of the four products would require four HPLC systems equipped with auto samplers and four analysts to finish the task of one robotic run, which would require only one person for half a day. The maximum capacity of the automated system is five lots of any combination or potency of the four products per run, after which a manual intervention to reload the system is needed.

Figure 2 shows a multi-application system based on a 'PY Technology' robot by Zymark. The system is designed for quality control (QC) testing of vitamins and is capable of performing liquid/liquid and solid/liquid extraction. Each application run can handle up to 36 samples of any combination of solid or liquid multivitamin formulations and raw materials. The generic design of this system is very flexible because it offers automated sample preparation and analysis of a variety of pharmaceutical dosage forms, e.g. solids, liquids, lotions, creams etc. If the chemistry requires protecting sample components from air, the system is equipped to purge each sample with nitrogen during various stages of preparation. A wide range of extracting temperatures is available through a special circulated water or oil bath. The choice of up to six extracting solvents is also available through two master lab stations.

Dissolution testing is becoming one of the most important single parameter to monitor for regulatory authorities. The volume of samples to be tested through various stages of product development and for release testing have increased the workload of analytical laboratories.

Manual dissolution is one of the most labour intensive and tedious tasks in the pharmaceutical testing laboratory, especially for sustained release formulations. This situation is a tremendous burden for laboratory personnel. Automating this process is highly recommended and should be one of the highest priorities for pharmaceutical laboratory managers.

The earlier developments in automating sampling and UV/HPLC analyses of some dissolution apparatus have been recently evolved to the fully automated robotic applications. The former apparatus consists of an IBM PC which controls dissolution baths (up to 24 vessels), delivery system and the UV/Vis spectrophotometer, or a fraction collector to collect samples for HPLC finish. An analyst is needed only to replace media and add samples to the vessels. This system is currently used by the Quality Control laboratory at the BMS New Brunswick facility

Examples of robotic-controlled dissolution systems are shown in figures 3 and 4. These are two of four systems which are used by the Analytical R&D group to perform dissolution of solid dosages. The systems are equipped with options of using baskets or paddles. The robot controls all the steps and parameters of a dissolution testing, including cleaning vessels, changing media and adding samples. Figure 5 shows the use of the robot to analyse pharmaceutical products and metabolites in biological fluids. *In vivo* bioavailability is an important parameter during the product development stage.

The system shown in figure 6 is designed to serve the radiopharmaceutical area. The parts of the robot that handle radioactive material are clad with lead sheets. The

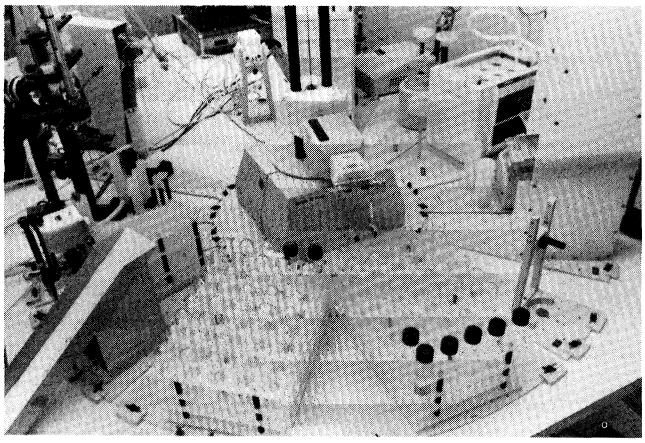

*Figure 2. A Zymark PY Technology robotic system for HPLC testing of multivitamin formulations.*

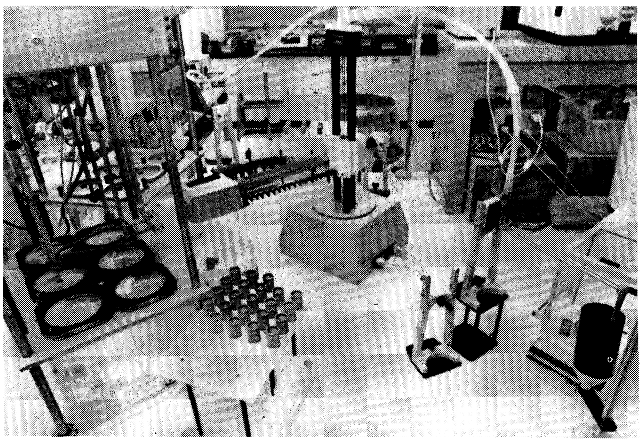

*Figure 3. A Zymark custom dissolution robot equipped with basket application of UV finish testing.*

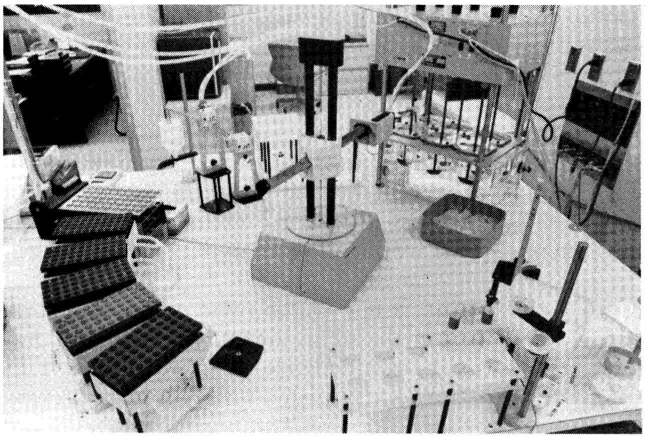

*Figure 4. A Zymark custom dissolution robot equipped with paddle application.*

robot adds and mixes the radioactive diluent and injects an aliquot into an HPLC system equipped with a gamma counter detector. Figure 7 represents a system which is used by the Quality Control laboratory for microbiological assay of neomycin by turbidimetric technique. The robot controls precise dilutions, additions, inoculation and incubation at 37 °C.

These successful applications are examples of a continued effort to automate laboratory functions within the BMS pharmaceutical group.

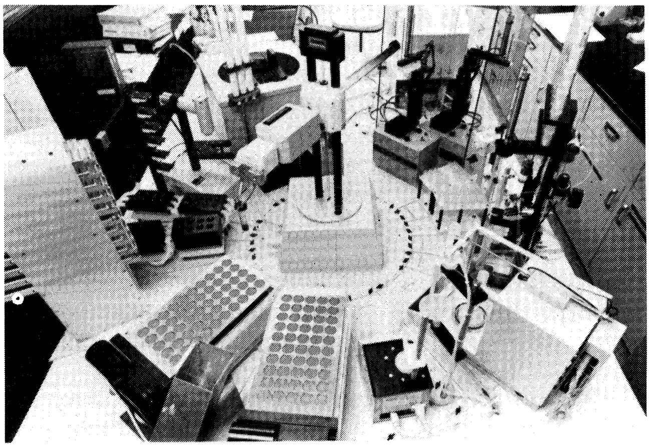

*Figure 5. A Zymark PY Technology robot for testing of biological samples.*

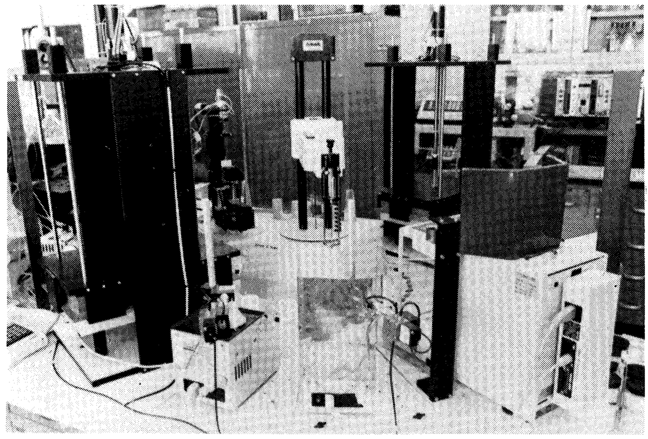

*Figure 6. A Zymark custom robot for radiopharmaceutical testing.*

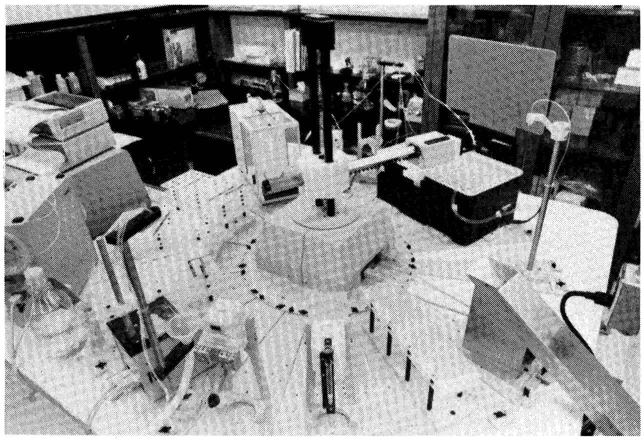

*Figure 7. A Zymark robotic system for microbiological testing.*

The laboratories involved provide various analytical services for chemical control, microbiological control, and analytical research and development. The dramatic increase in the number of robots in the New Brunswick facility, from one robot first acquired by the Quality Control department in 1985 to 22 by early 1990, is the result of dedication, hard work and continuous effort to face new challenges and new applications. This also reflects the commitment of BMS management to provide customers with high quality and effective products.

### Conclusion

The use of robots in analytical laboratories has been expanded to automate various laboratory functions. In order for laboratory managers to develop successful applications and increase efficiency and productivity, a few guidelines may be observed.

(1) Gaining the support and participation of laboratory personnel by demonstrating that expanding robotic technology is not going to pose a direct threat to their jobs. On the contrary, the new technology should be presented as new opportunities for advancement. It should be well known that the human contribution and innovation for any robotic applications will remain the main factor for the overall success.

(2) Vendor responsibilities for maintenance, repair, continuous updates of existing systems, and proper communications to provide application support to existing and future users will be the second most important factor in expanding the use of laboratory robotics.

(3) A few members of laboratory personnel that still doubt the reliability of laboratory robots should seriously reconsider their standing. Automation via robotics provides high-quality results including precision with good reproducibility. Consistency of everyday results is one of the most significant outcomes of robotics. Other known benefits include greater efficiency and productivity, high sample throughput, safety and GLPs.

### Acknowledgements

The authors wish to thank Mr R. Rowinski, Vice President, Bristol-Myers Squibb, North America Quality Control/Quality Assurance, and Dr J. Battaglia, Director of Quality Control, for their continuous support and encouragement. We would also like to extend our thanks to Mr R. Yura for his photography; Dr S. Conders, Dr M. Arnold, Ms D. Miller and Mr R. Fisco for allowing us to record their applications.

